# Evaluation of the validity and reliability of the Persian version of the refugee post-migration stress scale

**DOI:** 10.1186/s40359-025-03686-w

**Published:** 2025-12-10

**Authors:** Soore khaki, Fariba Hosseinzadegan, Abbas Ebadi, Seyed Qasem Mousavi, Amir Reza Tavakoli, Salman Barasteh

**Affiliations:** 1 Department of Medical -Surgical, School of Nursing & Midwifery, Shahid Beheshti University of Medical Science, Tehran, Iran; 2 Department of Medical-Surgical Nursing, School of Nursing and Midwifery, Tabriz University of Medical Sciences, Tabriz, Iran; 3 Nursing Care Research Center, Clinical Sciences Institute, Baqiyatallah University of Medical Sciences, Tehran, Iran; 4 Student Research Committee, Baqiyatallah University of Medical Sciences, Tehran, Iran

**Keywords:** Post-Migration stress, Refugees, Psychometrics, Validity, Reliability, Iran

## Abstract

**Background:**

Refugees usually face stressful events both in the destination country and during migration. To date, no tool has been designed to reflect the post-migration stress of Persian-speaking refugees on the basis of their current life conditions. Therefore, this study was conducted to determine the psychometric characteristics of the Persian version of the Refugee Post-migration Stress Scale (RPMS).

**Methodology:**

This cross-sectional study was conducted in 2022 with 355 Iranian refugees in Turkey. The original RPMS includes 21 items and 7 subscales. First, the original version of the tool was translated into Farsi via the standard forward-backward method. The instrument’s validity was checked through face validity, content validity, and construct validity via confirmatory factor analysis (CFA) and convergent validity with the World Health Organization-Five Well-Being Index **(**WHO-5) and the Hopkins Symptom Checklist 25 (HSCL-25(. Reliability was evaluated via the internal consistency method (Cronbach’s alpha). SPSS version 16 and LISREL version 8.8 software packages were used for data analysis.

**Results:**

Face validity and content validity were confirmed by refugees and experts with slight changes. CFA revealed that the proposed 7-subscale model of the original RPMS has a good fit (RMSEA: 0.08, NFI: 0.92; CFI: 0.95; IFI: 0.95; GFI 0.86; SRMR: 0.06). The convergent validity results also showed that the studied instrument has a suitable structure. Convergent validity was confirmed via the Pearson correlation results between the RPMS and two instruments: the HSCL-25 (*p* < 0.001, *r* = 0.33) and the WHO-5 (*p* < 0.001, *r*= -0.30). Additionally, the Cronbach’s alpha coefficient was 0.88.

**Conclusion:**

According to the psychometric results of the tool in the Persian-speaking refugee population, the tool is appropriate for measuring post-migration stress in refugees. Another advantage of this tool is its brevity and shortness.

## Introduction

Refugees often face traumatic experiences such as harassment, physical and sexual violence, and life-threatening situations both during migration and in the destination country [1, 2]. These traumatic events can result in long-term mental health consequences [3, 4]. Additionally, the living conditions for refugees are difficult in neighboring countries, and resettling in a socially and culturally unfamiliar country has additional stress [5]. This situation can lead to unemployment, loneliness, and uncertainty about asylum procedures and the future [6]. Other significant post-migration stressors specific to refugees include perceived discrimination, poor language and communication skills, separation from family, financial problems and unemployment, lack of private accommodation, social isolation, and loss of position. In addition, refugees often report poor social support and conflicts between spouses or parents and their children [7].

Stressful experiences during displacement have a direct negative effects on the mental health refugees [8, 9]. These effects are often intensified by socioeconomic disadvantage and poor living conditions [3, 4]. Refugees are at risk of developing long-term mental disorders, such as post-traumatic stress disorder (PTSD), anxiety, and depression [10, 11]. Some studies have reported that the prevalence of depression is between 5% and 80% and that PTSD rates are between 3% and 88% [10, 12, 13]. A study by Morina et al. confirmed this finding, revealing changes in mood and anxiety disorders, alcohol dependence and psychosis [14]. Furthermore, Byrow et al. confirmed a relationship between post-migration stress and mental health among refugees [15].

Several instruments are commonly used to assess post-migration stress. The Harvard Trauma Questionnaire, which is used to assess trauma-related stress in refugees, is a scale that assesses the effects of torture, trauma, and PTSD [16]. The Hopkins Symptom Checklist-25 measures symptoms of anxiety and depression, which are common among refugee populations experiencing post-migration stress [17]. The Demands of Immigration (DI) Scale assesses general stressors associated with immigration, such as language difficulties and cultural adjustment [18]. MIGSTR10 (Migration-Related Stress) assesses stressors related to migration and acculturation in patients with mental disorders and a history of migration [19]. However, these scales have not been specifically designed to refugees. They typically focus on general immigrant experience and fail to capture the unique, refugee-specific stressors, such as forced discrimination, financial strain, or family separation. Furthermore, these scales are not specifically designed for refugees and are not specifically designed to capture refugee-specific experiences.

It is essential to distinguish between refugees and immigrants when assessing post-migration stress. Refugee-specific stress is unique and includes challenges of adapting to a new country, forced displacement, exposure to life-threatening violence, and long-term uncertainty in asylum, which are usually rarely experienced by voluntary immigrants [20–22]. To address these limitations the Refugee Post-Migration Stress Scale (RPMS) was designed specifically for the refugee populations. The RPMS seven subscales address aspect of refugee life such as perceived discrimination, family separation, and material strain. This scale is particularly notable for addressing the complex post-migration stressors that are specific to refugees [5], whereas instruments such as the DI [18] and MIGSTR10 [19] are more general and appropriate for broader groups of migrant populations. This tool has been used in some countries, such as Sweden, Norway and Turkey [7, 23–25]. However, to date, no validated instrument exists to evaluate post-migration stress experienced by Persian-speaking refugees. Therefore, the present study aims to translate and evaluate the psychometric properties of the Persian version of the RPMS among Iranian refugee populations.

## Methodology

### Study design

This cross-sectional study was conducted on a population of Iranian refugees in Turkey. Data were collected from October to December 2022. The inclusion criteria for participation were as follows: willingness to participate in the study, being literate in reading and writing and speaking in Persian, not having a history of psychological disorders on the basis of self-reports, being at least 15 years old, not participating in another study with the theme of post-migration stress, and providing informed consent to participate in the study. The exclusion criteria included not returning the questionnaire and incomplete completion of the scale.

### Study participants and sampling methods

Convenience sampling was employed to select the participants. Refugees were recruited from Yalva and Istanbul across Turkey. To gather data, the first author of the study personally visited nongovernmental associations in both cities. In conducting confirmatory factor analysis (CFA), Klein [26] and Worthington & Whittaker [27] mentioned that a minimum sample size of 200 is appropriate for robust model evaluation [28], whereas CFA recommends 10 to 20 participants per item [29]. The questionnaires were distributed to 455 refugees, and 355 questionnaires were ultimately completed and returned. The response rate in this survey was 78%. To minimize potential biases in the sampling process, we took several steps: we ensured that the participants were informed about the study’s purpose and that their participation was voluntary. Additionally, we aimed to include a diverse range of refugees to increase the representativeness of our sample. However, we acknowledge that the use of convenience sampling may limit the generalizability of our findings.

### Study instruments

#### Demographic information questionnaire

This information included age, sex, marital status, education level, migration or refugee history and type of status.

#### The refugee post-migration stress scale (RPMS)

The RPMS tool was developed by Malm et al. (2020). This tool was developed to investigate the post-migration stress of Syrian refugees in Sweden. The seven dimensions of this tool include perceived discrimination, lack of host country-specific competencies, material and economic strain, loss of home country, family and home country concerns, social strain, and family conflicts. The face validity and content validity of the scale of the tool were examined. In addition, its construct validity was examined via exploratory factor analysis (EFA) and CFA. The answers to the items of the tool are scored on a 5-point Likert scale as follows: never (= 1), rarely (= 2), sometimes (= 3), a lot [4], and very much (= 5). The range of scores obtained from this questionnaire is between 21 and 105 [5].

#### HSCL-25 questionnaire

The short form of the SCL-25 was designed in Iran in 2001, and its psychometric properties were investigated. This version was revised in 2016 for its construct validity. The Persian version was prepared through the EFA. The participants’ responses on a Likert scale included never (= 0), a few (= 1), somewhat (= 2), great (= 3), and very great (= 4) according to the original scale. Higher scores indicate more psychological damage. Najarian and Davodi evaluated its validity through factor analysis, convergent and divergent validity, and reliability through internal consistency and test‒retest methods. They reported that the Cronbach’s α of the new version was 0.97 for women and 0.98 for men. In addition, the test‒retest coefficient after 5 weeks was 0.78 for the total sample, 0.77 for women, and 0.79 for men [27, 28]. The Cronbach’s α of the present study was 0.92.

#### World health organization-five well-being index (WHO-5-P)

The WHO-5-P is a positively worded instrument designed to assess the level of emotional well-being over a 14-day period. This tool had acceptable internal consistency (α = 0.94), and it has good convergent validity with the GHQ-28 (*r* = 0.66; *P* < 0.001). Additionally, the one-dimensional EFA included five items with positive wording, where the presence of positive emotions in the last 2 weeks was scored on a 6-point Likert scale from 0 (absent) to 5 (constantly present). Here, high scores indicate an increased sense of well-being, whereas a score below 13 indicates poor well-being [29]. In a study by Omani-Samani et al., CFA confirmed the unidimensional factor structure of the scale. Additionally, the internal consistency of the scale was 0.85 [23]. The Cronbach’s alpha of the present study was 0.83.

### Translation procedure

The translation process was performed via the Brislin double translation-back translation model [30]. The scale was translated to Persian via a systematic process that included the following steps: (1) Forward translation: two bilingual experts in both Persian and English independently translated the original scale into Persian. They were familiar with the context of psychology and the specific terminology used in the scale. (2) Synthesis of translations: the two translations were then compared, and any discrepancies were discussed among the translators to reach a consensus on a single Persian version. This step ensured that the nuances of meaning were preserved. (3) Back translation: The consensus Persian version was subsequently translated back into English by two other bilingual experts who had no prior knowledge of the original scale. This back-translation was essential for identifying any inconsistencies or loss of meaning in the translated version. (4) Cultural adaptation: Following back-translation, we conducted a review with a panel of experts in psychology and migration (Table 1). They provided feedback on the cultural relevance of the items and suggested modifications to ensure that the scale was appropriate for the Persian-speaking population. The English and Persian versions of the RPMS are presented in Table 2.

**Table 1:** Characteristics of the expert panel of the study participants

Participant No	Age	Sex	Experience (Years)	Degree	Specialization Field
Expert 1	56	female	17	Psychiatric specialist	mental health
Expert 2	63	male	25	PhD in psychology	Psychology
Expert 3	35	male	10	PhD in nursing	Nursing, expertise scale development
Expert 4	37	female	8	PhD in psychiatric nursing	Nursing, psychiatric nursing
Expert 5	42	Female	10	Master’s degree	Refugee studies
Expert 6	40	male	12	Master’s degree	Public Health

**Table 2:**
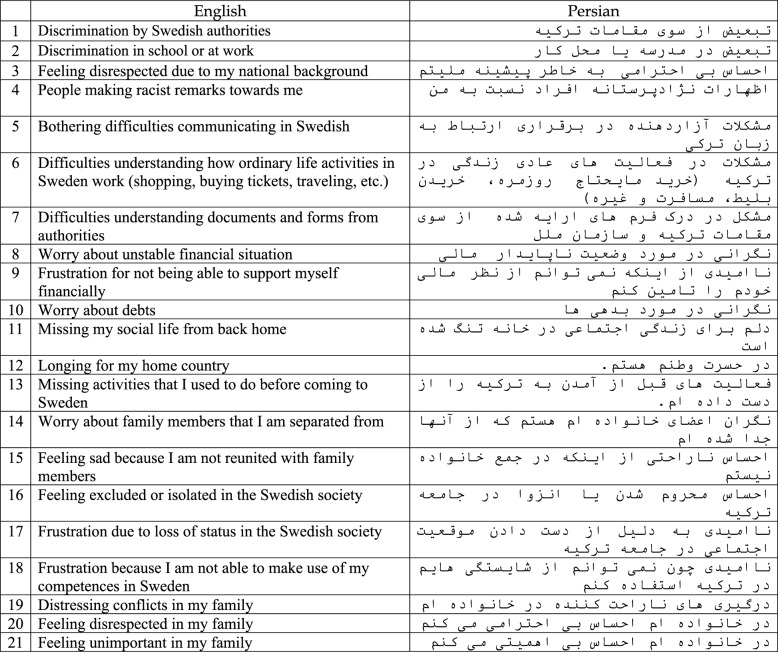
English and Persian versions of the RPMS

### Item analysis

At this stage, the goal of item analysis is to develop a targeted set of items for each dimension of the construct. A correlation coefficient below 0.3 suggests a weak relationship with the construct [31]. In this section, item analysis was conducted with 30 refugee participants via the loop method, and the results were analyzed via SPSS software, with Cronbach’s alpha also calculated. All the items had correlations above 0.3.

### Psychometric assessment of the scale

In this study, we examined the psychometric properties of the instrument, including content validity, item analysis, CFA, convergent validity, and internal consistency reliability.

### Face and content validity

After the translation process was complete, qualitative face validity was evaluated. The face validity of the scale was assessed by refugees, who are potential users. This approach helps identify potential errors by exploring respondents’ thought processes while completing the scale [32]. To assess the difficulty, suitability, and relevance of the items, face-to-face interviews were held with 10 asylum seekers. Content validation was performed to explore all important aspects of the intended concept of the instrument as well as the acceptance of execution and totality of the instrument by experts [31]. To examine content validity, the Persian version of the inventory was given to 10 specialists in palliative care, and they were asked to examine the relevancy of the items through a four-point Likert scale. Ultimately, the CVI score was calculated for the items. A content validity index greater than 0.79 is considered suitable, 0.7–0.79 needs correction and revision, and a score less than 0.7 is unacceptable and should be omitted [33].

### Ceiling and floor effects

Floor and ceiling effects occur when a considerable proportion of respondents endorse the best or worst score [34]. The measure is then unable to discriminate between respondents at either extreme of the scale. This may indicate a lack of content validity and responsiveness as well as reduced reliability because changes cannot be measured [35]. When more than 15% of participants acquire the maximum or minimum achievable score, it is called the ceiling and floor effect.

### Confirmatory factor analysis (CFA)

If the factor loading is less than 0.3, the correlation between the factor and the item is considered weak, and it is better to delete the item, as it cannot explain the variable well [36]. CFA is a technique used to determine the maximum likelihood for evaluating construct validity [28]. We used the maximum likelihood estimation method for the CFA. A total of 355 refugees were included. The analysis was conducted via LISREL (version 8.8), which employs various indicators to assess the model’s effectiveness. Model fit indices in CFA are categorized into three main types: absolute fit, which includes the root mean square error of approximation (RMSEA), standardized root mean square residual (SRMR), goodness-of-fit index (GFI), and chi-square; comparative fit, which encompasses the comparative fit index (CFI), incremental fit index (IFI), relative fit index (RFI), normed fit index (NFI), and Tucker–Lewis index (TLI); and parsimonious fit, which includes the parsimonious comparative fit index (PCFI), parsimonious normed fit index (PNFI), adjusted goodness-of-fit index (AGFI), and Akaike information criterion (AIC).

### Reliability

Reliability is defined as the consistency of a method in measuring something. A measurement is considered reliable if the same result can be consistently attained by applying the same methodology under similar conditions [37]. The reliability of the Persian version of the Refugee Post-Migration Stress Scale was measured via the internal consistency method (Cronbach’s alpha coefficient). Internal consistency reliability refers to the consistency within the measurement itself, meaning it assesses whether similar results are obtained from different parts of a test designed to measure the same construct [37]. By convention, an alpha of 0.65–0.80 is often considered “adequate” for a scale used in human dimension research [38].

### Data analysis

For data analysis, SPSS 26 was used along with the relevant tests together with LISREL 8.8 software. Descriptive statistics, including frequencies/percentages, means ± standard deviations, and analytical statistics, including parametric tests, correlations, and Cronbach’s alpha coefficients, were used. The normality of the data was assessed via the Kolmogorov‒Smirnov test. Additionally, a significance level of 0.05 was considered.

### Ethical consideration

The study was conducted after obtaining permission from the Ethics Committee of Baqiyatullah University of Medical Sciences with the code IR.BMSU.REC.1400.093. The translation process was performed after written permission was obtained via email from the tool developer. Verbal and written informed consent was obtained from the participants according to the criteria of Helsinki et al. The participants were also informed that the research data would remain confidential and that they could leave the study anytime.

## Results

### Demographic status

This research investigated 355 refugees with an average age of 32.72 ± 7.60 years who had immigrated to Turkey. Approximately 58.3% of the patients were male, and 41.7% were female. Additionally, 36.3% were married, and 32.7% were single. In terms of education, 60.3% had a university education, and 39.7% did not have a university education. Table 3 shows the demographic characteristics of the participants.

**Table 3:** Demographic characteristics

Variable	*n*	%	Statistical test	Results
sex				
Male	148	58.3	Independent T test	*P* < 0.001 T = 54.07
Female	207	41.7		
Age(Mean ± SD (32.72 ± 7.60				
15–20	19	5.4	One way ANOVA	*P* < 0.001 F = 5.96
21–40	288	81.1		
> 40	48	13.5		
Marital				
Single	116	32.7	One way ANOVA	*P* = 0.03 F = 3.41
Married	129	36.3		
Divorce/widow	110	31.0		
Education				
Up to Diploma	141	39.7	One way ANOVA	*P* < 0.001 F = 7.99
BS	180	50.7		
MS & PhD	34	9.6		
Migration or Refugee History (years ± SD) 4.10 ± 2.60				
1–2	109	30.7	One way ANOVA	*P* = 0.27 F = 1.28
2–5	155	43.7		
> 5	91	25.6		
Type of status				
Migration	89	25.1	Independent T test	*P* = 0.21 T = − 1.24
Refugee	266	74.9		

To assess the normality of the data, the Kolmogorov‒Smirnov test was conducted, and the *p* value was found to be greater than 0.05. Therefore, parametric tests were used. There was a statistically significant relationship between sex (*P* < 0.001), marital status (*P* = 0.03), education level (*P* < 0.001), and RPMS. The mean and standard deviation of the RPMS was 60.12 ± 12.50. Moreover, the mean scores of the RPMS subscales ranged from 9.06 to 12.3. The highest mean was related to perceived discrimination (12.3 ± 3.96), and the lowest was related to the lack of host country-specific competences (9.06 ± 2.54) and social strain (9.06 ± 2.35) (Table 4).

**Table 4:** The means and standard deviations of the scores of each of the RPMS subscales

Factor	Item No.	Minimum	Maximum	Mean(SD)
Perceived discrimination	4	4	20	12.3(3.96)
Lack of host country specific competences	3	3	15	9.06(2.54)
Material and economic strain	3	3	15	9.64(2.34)
Loss of home country	3	3	15	9.67(2.37)
Family and home country concerns	2	2	19	6.51(2.26)
Social strain	3	3	15	9.06(2.35)
Family conflicts	3	3	15	7.17(3.22)
TOTAL RPMS	21	20	100	60.12(12.50)

### Translation procedure

After completing the translation and approval of the translated version of the questionnaire by Malm, the Persian questionnaire included 21 items and seven main subscales.

### Face and content validity

The face validity of the scale was evaluated qualitatively with the participation of 10 asylum seekers by assessing the clarity, relevance, and simplicity of the items; no ambiguities or issues were identified. For content validity, the content validity index (CVI) was calculated for each item, with all items scoring above 0.79. The item-level CVIs (I-CVIs) ranged from 0.8 to 1, indicating acceptable content validity; therefore, no items were excluded at this stage. Given the simplicity and clarity of the items, only minor revisions were made to enhance content validity.

### Construct validity

The construct validity of the proposed scale was determined via two methods: CFA and convergent validity. A KMO value of 0.883 was found, and Bartlett’s test of sphericity was significant (X2 = 3524.156513, df = 210, *p* < 0.001) for the scale. In this study, the loading factor for the items was greater than 0.35 (Table 5).

**Table 5:** Factor loading of the RPMS

Factor	Items	Factor Loading %
Perceived discrimination	RPMS1	0.69
	RPMS2	0.74
	RPMS3	0.69
	RPMS4	0.67
Lack of host country specific competences	RPMS5	0.62
	RPMS6	0.53
	RPMS7	0.52
Material and economic strain	RPMS8	0.53
	RPMS9	0.35
	RPMS10	0.43
Loss of home country	RPMS11	0.40
	RPMS12	0.49
	RPMS13	0.35
Family and home country concerns	RPMS14	0.63
	RPMS15	0.60
Social strain	RPMS16	0.53
	RPMS17	0.45
	RPMS18	0.40
Family conflicts	RPMS19	0.65
	RPMS20	0.72
	RPMS21	0.71

### CFA

**Fig. 1:**
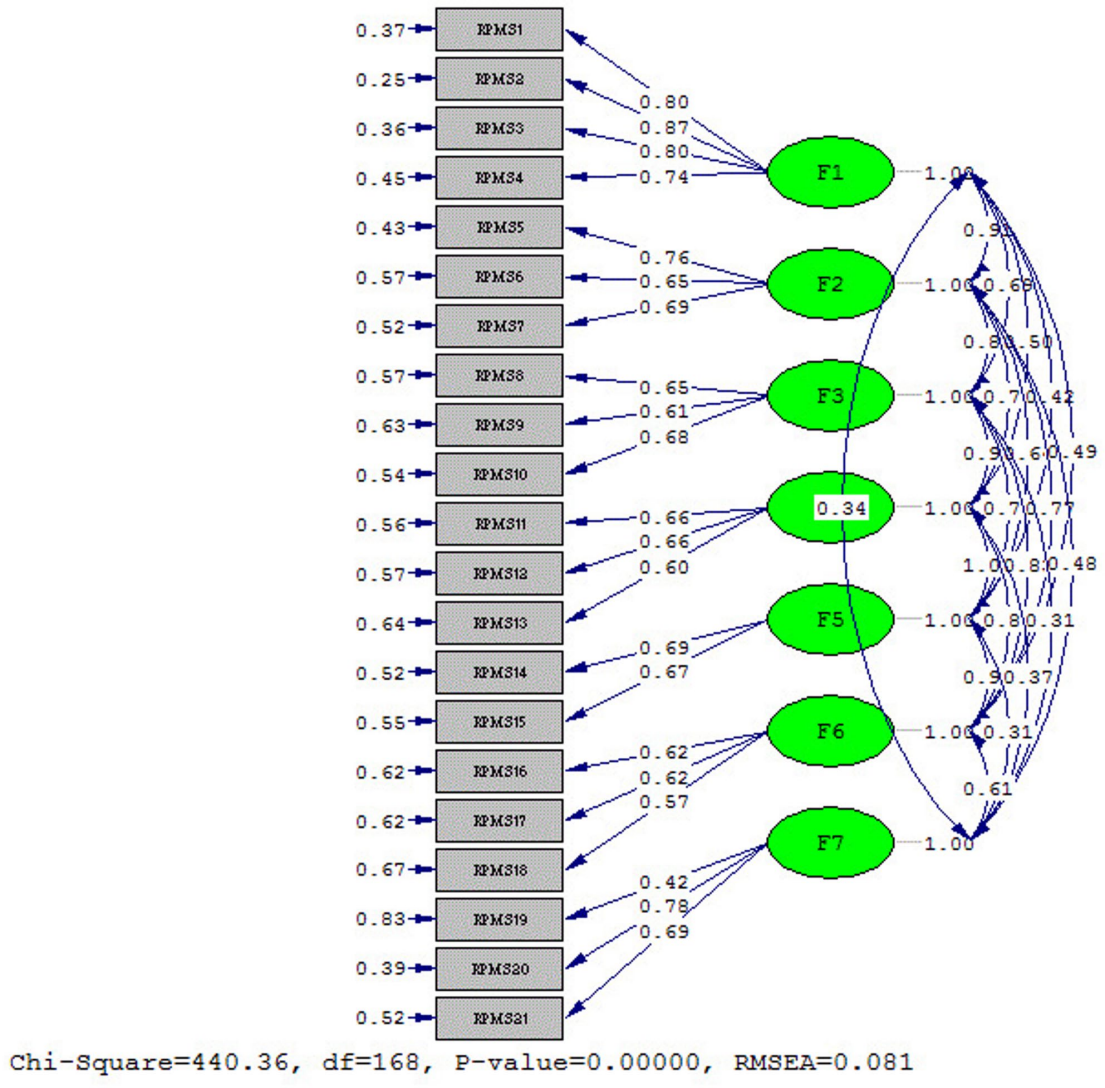
Final structure of the model

The model had a good fit in the CFA. The examined goodness-of-fit indices were as follows: normed fit index (NFI) = 0.91, root mean square error of approximation (RMSEA) = 0.08, goodness-of-fit index (GFI) = 0.86, standardized root mean square residual (SRMR) = 0.069, comparative fit index (CFI) = 0.95, and incremental fit index (IFI) = 0.91. The results of the CFA are presented in Table 6 and Fig. 1.

**Table 6:** Confirmatory factor analysis (CFA) results

Fit Indices	Normal Range	Result
*P* value, Chi-squared	0.05<	0.00, 440.36
RMSEA (Root Mean Square Error of Approximation)	good < 0.08, average < 0.08–0.1, weak < 0.1	0.08
SRMR (Standardized Rroot Mean Square Residual)	1/0>	0.069
PNFI (Parsimonious Normed Fit Index)	0.5<	0.74
NFI (Normed Fit Index)	0.9<	0.92
AGFI (Adjusted Goodness-of-Fit Index)	0.8<	0.80
GFI (Goodness-of-Fit Index)	0.9<	0.86
(RFI (Fit Index Relative	0.9<	0.91
IFI (Index Tucker‒Lewis)	0.9<	0.95
TLI (Fit Index Relative)	0.9<	0.91
CFI (Comparative of Fit Index)	0.9<	0.95
CMIN/DF (Minimum Discrepancy Function by Degrees of Freedom divided)	good < 3 Acceptable < 5	2.61

### Convergent validity

For convergent validity, the results revealed a statistically significant correlation between the total scores of the Persian version of the RPMS with the Hopkins Symptom Checklist 25 (HSCL-25) (*r* = 0.33, *p* = 0.01) and the WHO-5 Well-being Index (WHO-5) (*r* = 0.30, *p* < 0.001).

### Reliability

In terms of scale reliability, the internal consistency according to the Cronbach’s alpha coefficient for the subscales of the scale ranged from 0.53 to 0.88, and that for the total RPMS was 0.88 (Table 7).

**Table 7:** Reliability of the RPMS and its subscales

Factor	Cronbach’s Alpha
Perceived discrimination	0.88
Lack of host country specific competences	0.77
Material and economic strain	0.56
Loss of home country	0.53
Family and home country concerns	0.78
Social strain	0.66
Family conflicts	0.83
TOTAL RPMS	0.88

## Discussion

The findings of the present study provide strong evidence for the psychometric robustness of the Persian version of the RPMS, demonstrating its suitability for assessing post-migration stress among Persian-speaking refugees. The scale shows appropriate face validity and content validity were confirmed by participants and experts. Additionally, the results of construct validity, as confirmed by CFA and convergent validity. The scale reliability was approved by the internal consistency (Cronbach’s alpha coefficient = 0.88).

Compared to other tools assessing refugee stress, such as the Harvard Trauma Questionnaire (HTQ) [16], the Demands of Immigration Scale (DIS) [18], and the Hopkins Symptom Checklist-25 (HSCL-25) [17], the RPMS provides a comprehensive evaluation of stressors of post-migration including social, relational, and support-related stressors [5]. Its brevity further enhances its practical value for clinical use, allowing mental health professionals and policymakers to efficiently screen for post-migration stress and design appropriate interventions.

The main findings include confirmation of face validity and content validity through feedback from refugees and experts, as well as strong construct validity through CFA. The Persian version of the RPMS instrument shows good internal consistency (Cronbach’s alpha = 0.88), indicating its reliability for use in clinical settings. These results are consistent with those of previous studies, such as Malm et al.‘s study on the original RPMS (α = 0.86) [5] and Alexander et al.‘s work on Syrian refugees (α = 0.80–0.84), indicating similar reliability across cultural contexts [7].

The Persian version of the RPMS was carefully translated, and the clarity and simplicity of the items were ensured on the basis of feedback from 10 refugees. This confirms the face validity of the instrument, similar to the findings of the study by Malm et al., who assessed the face validity of the original RPMS with 7 Iraqi refugees in Sweden [5]. Content validity was established through expert feedback, with six experts confirming the accuracy and relevance of the items in the original version, similar to the procedure used in this study. Both studies demonstrated strong content validity, with the original version having an I-CVI between 0.8 and 1 and an S-CVI of 0.95 [2].

The construct validity of the Persian version of the scale was confirmed by CFA, in which a seven-dimensional model of the original version of the RPMS was confirmed in Persian. The fit indices revealed a good model such that the RMSEA was acceptable (RMSEA = 0.08). This finding is consistent with Tatlioglu et al.’s study on Syrian refugees, where the CFA also showed a good fit for the RPMS model, and the RMSEA value ​​was 0.072 before and 0.066 after the modification [25]. These results demonstrate the strong construct validity of the instrument and confirm its potential for use in Persian refugee populations with different cultural backgrounds.

The convergent validity of the Persian version of the RPMS was confirmed through significant correlations with the HSCL-25 and the WHO-5. A moderate positive correlation was found between the RPMS score and the HSCL-25 score (*r* = 0.33, *p* < 0.001), which indicates a relationship between post-migration stress and mental health symptoms. Furthermore, a negative correlation was found with the WHO-5 score (*r* = −0.30, *p* < 0.001). These findings are consistent with those of previous studies by Malem [5] and Tatlioglu [25] and emphasize the convergent validity of the instrument. Although the correlations are statistically significant, they are relatively small. The multifaceted nature of post-migration stress, as well as factors such as individual resilience, social support, and contextual variables, likely plays important roles and may moderate the magnitude of these associations. Furthermore, differences in the constructs measured by these scales—the RPMS focusing on migration-specific stressors, the HSCL-25 on general psychological distress, and the WHO-5 on subjective well-being—may also contribute to the moderate correlations observed. Thus, while these findings support the convergent validity of the RPMS, they also highlight the complexity of post-migration stress and its relationship with broader mental health outcomes.

Internal consistency for the total scale was high (α = 0.88). This is support its reliability, and which is consistent with the original RPMS (Cronbach’s alpha = 0.86) [5]. The findings of the present study are consistent with the findings of Alexander’s study, with a Cronbach’s alpha of 0.84 [7], and Dangman’s study, with a Cronbach’s alpha of 0.77 [24]. In social science research, an alpha of 0.65 or 0.7 or higher is usually considered acceptable. In this study, two subscales, namely, “Material and economic strain” (0.56) and “Loss of home country” (0.53), fall below this conventional threshold. Alphas below 0.65 or 0.7 indicate lower internal consistency, which may be due to items in these subscales not being highly correlated, a small number of items in the subscale [39], heterogeneity of item content [40], or variability in respondents’ understanding [41]. There is less internal consistency since various cultural groups experience and report constructs like economic distress or loss of homeland in different ways. This emphasizes how crucial cultural sensitivity is when creating and improving tools for assessing refugees [42]. Future studies should investigate the potential effects of economic norms, hometown values, and emotional expression on item performance on these subscales.

Cultural differences, including psychological barriers related to social stigma toward mental health issues, collectivist values emphasizing family and community, and the diverse immigration experiences of Persian-speaking immigrants, can influence how psychological stressors—such as economic hardship and loss of homeland—are perceived and reported. These cultural factors may contribute to the observed reduction in Cronbach’s alpha for subscales addressing these dimensions, underscoring the need for further culturally sensitive research and adaptation of measurement tools [43, 44]. Future research could use qualitative methods with the participation of community stakeholders to enhance the validity and cultural relevance of this scale and clarify these subtle cultural differences.

## Conclusion

The psychometric results of the instrument in the Persian sample indicate that it is efficient for measuring post-migration stress in refugees. Another advantage of the tool is its brevity and shortness. Therefore, clinicians and therapists can use it in their routine assessments to identify and monitor post-migration stress. Policymakers working with refugees can also use this tool to examine the extent of post-migration stress and evaluate the effectiveness of social interventions. Additionally, researchers can use this tool to more accurately investigate the psychological factors associated with post-migration stress.

### Limitations

One of the major limitations of the current study, similar to most cross-sectional studies, is the selection of participants on the basis of a convenience sampling method, necessitated by special study conditions in another country. This approach resulted in a sample consisting solely of Iranian Farsi speakers abroad, making the generalizability of the findings to non-Iranian Farsi speakers ambiguous. Additionally, the relatively small sample size may limit the statistical power and generalizability of the results to broader populations. Methodological constraints, such as reliance on self-reported data, could introduce biases related to social desirability or recall inaccuracies, further affecting the validity and applicability of the findings across different contexts. Moreover, owing to limited access to participants for revisiting, only internal consistency was used to check the instrument’s reliability, precluding test‒retest reliability assessments. The study’s focus on a specific demographic may restrict the ability to extrapolate findings to other groups or settings. Future research should aim to address these limitations by utilizing larger, more diverse samples and employing mixed methods to triangulate data. Exploring these avenues will increase the robustness of the findings and contribute to a more comprehensive understanding of the topic within the field.

## Data Availability

All the data generated or analyzed during this study are included in this published article.

## Abbreviations

RPMS: Refugee post-migration stress scale
PTSD: Post-traumatic stress disorder
MIGSTR10: Migration and acculturation-related stress
HTQ: Harvard Trauma Questionnaire
WHO: World Health Organization
HSCL-25: Hopkins Symptom Checklist-25
EFA: Exploratory factor analysis
CFA: Confirmatory factor analysis
KMO: Keiser-Meyer-Olkin
S-CVI: Scale-content validity scale
IQOLA: International Quality of Life Assessment
RMSEA: Root mean square error of approximation
NFI: Normal fit index
CFI: Comparative fit index
IFI: Incremental fit index
GFI: Goodness-of-fit index
Standardized RMR: Standardized root mean square residual
